# Deletions on 9p21 are associated with worse outcomes after anti-PD-1/PD-L1 monotherapy but not chemoimmunotherapy

**DOI:** 10.1038/s41698-022-00286-4

**Published:** 2022-06-23

**Authors:** Ericka M. Ebot, Daniel L. Duncan, Khaled Tolba, David Fabrizio, Garrett M. Frampton, Leah A. Comment, Lee A. Albacker

**Affiliations:** 1 Foundation Medicine, Inc., Cambridge, MA USA; 2 Foundation Medicine, Inc., Morrisville, NC USA

**Keywords:** Non-small-cell lung cancer, Tumour biomarkers, Cancer immunotherapy, Cancer genomics

## Abstract

NCCN guidelines for first-line treatment of advanced non-squamous non-small-cell lung cancer (NSCLC) patients without targetable driver alterations includes either immunotherapy alone or in combination with chemotherapy. In this study, we investigated genomic predictors of survival after immunotherapy to guide this treatment decision. Cox proportional hazards regression was used to identify genomic correlates of survival in a cohort of *EGFR*/*ALK*-, non-squamous NSCLC patients treated with first-line pembrolizumab monotherapy (mono-IO) or pembrolizumab in combination with carboplatin/cisplatin and pemetrexed (chemo-IO) within a real-world clinico-genomic database. The effect of deletions on 9p21 was further evaluated in five additional tumor types. Among mono-IO treated non-squamous NSCLC patients, tumors with 9p21.3 gene deletions (*CDKN2A*, *CDKN2B*, *MTAP*) were associated with worse survival compared to the corresponding deletion-negative tumors (*CDKN2A* deletion HR = 1.8, *P* = 0.001). However, this association was not observed among chemo-IO treated patients (*CDKN2A* deletion HR = 1.1, *P* = 0.4). This finding remained after adjusting for clinical and genomic features including TMB and PD-L1. Deletions at 9p21.3 were not associated with differences in TMB, PD-L1, or tumor inflammation. Due to the high incidence of 9p21.3 deletions across tumor types, we performed a pan-cancer analysis and found *CDKN2A* deletion-positive tumors had worse survival following first-line immunotherapy treatment in multiple tumor types (HR = 1.4, *P* < 0.001). These results indicate deletions at 9p21.3 are a putative negative predictor of clinical benefit from first-line immune checkpoint inhibitors and may have utility in choosing between mono-IO vs chemo-IO regimens in NSCLC.

## Introduction

Immune checkpoint inhibitors (ICIs) have transformed clinical care of cancer patients. Since the first approval in 2015, ICI therapy targeting PD-1/PD-L1 has been established as the standard of care for patients with advanced non-small-cell lung cancer (NSCLC)^1^. The KEYNOTE-024/042 and KEYNOTE-189 clinical trials established the clinical utility of checkpoint inhibitor blockade as a monotherapy and in combination with chemotherapy in the first-line setting for patients with advanced non-squamous NSCLC that lack targetable alterations^2–4^. While both ICI monotherapy and ICI chemotherapy combination are recommended first-line treatments these treatments have not been directly compared in a clinical trial.

Tumor PD-L1 expression, tumor mutational burden (TMB), and microsatellite instability are FDA-approved biomarkers for anti-PD-1 therapy^5^. While each of these biomarkers enrich for improved patient outcomes, a significant number of biomarker negative patients respond to therapy. A better understanding of the molecular determinants of immunotherapy response is needed to identify patients who will benefit from ICI treatment and to guide treatment decisions between ICI monotherapy and chemo combination regimens for advanced non-squamous NSCLC patients.

To overcome the knowledge gaps left by clinical trials, we utilized a real-world clinico-genomic database (CGDB) to compare outcomes of advanced non-squamous NSCLC patients treated with mono-immunotherapy versus chemo-immunotherapy. We identified deletions at the 9p21.3 locus (*CDKN2A, CDKN2B,* and *MTAP*) as negative genomic predictors of survival to single-agent immunotherapy. In lung adenocarcinoma specimens, no relationship was observed between *CDKN2A* deletion and immune-inflamed phenotype, unlike PD-L1, suggesting it may be a mechanistically distinct predictor of checkpoint inhibitor efficacy. Finally, we extended the NSCLC findings by demonstrating an association between *CDKN2A* deletion and survival in ICI-treated patients across multiple cancer types.

## Results

### Non-squamous NSCLC patient characteristics

**Fig. 1:**
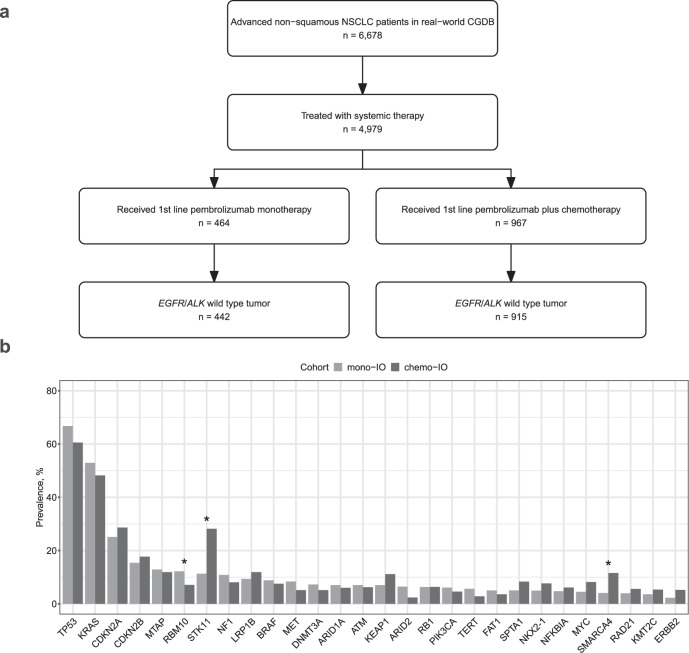
Clinical cohort selection and genomic characterization. **a** Flowchart depicting clinical cohort selection. The NSCLC CGDB included 6678 patients who had a medical record-confirmed diagnosis of advanced non-squamous NSCLC, received care within the Flatiron Health network between January 2011 and December 2020, and underwent tissue-based CGP by Foundation Medicine during their cancer care. Among these patients, 4979 had documentation of initiation of at least one line of systemic therapy after advanced diagnosis. This study was limited to advanced non-squamous NSCLC patients whose tumor did not harbor an *EGFR* short variant or *ALK* rearrangement, and received first-line treatment with pembrolizumab monotherapy or first-line treatment with pembrolizumab plus pemetrexed and platinum-based chemotherapy. **b** Bar chart of the prevalence of gene alterations by treatment cohort. *FDR *p*-value < 0.05.

This study included advanced non-squamous NSCLC patients without *EGFR* mutations or *ALK* rearrangements (*EGFR*/*ALK*-) treated with first-line pembrolizumab monotherapy between 2016 and 2020 (mono-IO cohort, *n* = 442) or first-line pembrolizumab plus chemotherapy between 2017 and 2020 (chemo-IO cohort, *n* = 915) within the real-world CGDB (Fig. 1a). Patients in the mono-IO cohort were older (median age: 72 years vs 68 years), more likely to be female (57% vs 47%), and more likely to have a history of smoking (95% vs 90%) compared to patients in the chemo-IO cohort (Table 1). A higher proportion of mono-IO treated patients had non-advanced stage at initial diagnosis (32% vs 16%) and poor ECOG PS (≥2: 24% vs 16%) compared to chemo-IO treated patients. 49% of patients in the mono-IO cohort had tumors with high TMB (≥10 mut/Mb) compared to 36% of patients in the chemo-IO cohort. Almost all (93%) mono-IO treated patients had PD-L1-positive (PD-L1+) tumors, compared to 62% of chemo-IO treated patients. Median overall survival (mOS) for the mono-IO and chemo-IO cohort was 15.7 [13.4–22.3] months and 12.3 [10.5–14.0] months respectively.

**Table 1:** Demographic and clinical characteristics by treatment cohort.

	1st line mono-IO (*n* = 442)	1st line chemo-IO (*n* = 915)	*P*-value^a^
Age at treatment start, years, median (range)	72 (38, 85)	68 (33, 85)	<0.001
Gender, *n* (%)			0.001
Female	251 (57)	427 (47)	
Male	191 (43)	488 (53)	
Race, *n* (%)			0.1
Asian	5 (1)	11 (1)	
Black or African American	19 (4)	60 (7)	
Other	65 (15)	144 (16)	
White	322 (73)	610 (67)	
Missing	31 (7)	90 (10)	
Smoking history, *n* (%)			0.004
Yes	418 (95)	822 (90)	
No	24 (5)	93 (10)	
Practice type, *n* (%)			0.3
Academic	31 (7)	50 (5)	
Community	411 (93)	865 (95)	
Advanced stage at diagnosis^b^, *n* (%)			<0.001
Yes	292 (66)	754 (82)	
No	143 (32)	150 (16)	
Not reported	7 (2)	11 (1)	
ECOG performance status^c^, *n* (%)			<0.001
0	88 (20)	251 (27)	
1	163 (37)	332 (36)	
2+	107 (24)	144 (16)	
Missing	84 (19)	188 (21)	
Tumor type, *n* (%)			0.02
Lung adenocarcinoma	346 (78)	773 (84)	
Lung non-small cell lung carcinoma (nsclc) (nos)	64 (14)	93 (10)	
Other	32 (7)	49 (5)	
Tissue of origin, *n* (%)			0.3
Lung	211 (48)	426 (47)	
Lymph node	60 (14)	125 (14)	
Brain	37 (8)	52 (6)	
Bone	19 (4)	55 (6)	
Other/unknown	115 (26)	257 (28)	
TMB, *n* (%)			<0.001
Low (<10 mut/Mb)	227 (51)	583 (64)	
High (≥10 mut/Mb)	215 (49)	332 (36)	
PD-L1 expression, *n* (%)			<0.001
No (<1% TPS)	16 (7)	184 (38)	
Low (1–49% TPS)	33 (15)	170 (36)	
High (≥50% TPS)	174 (78)	124 (26)	
Missing	219	437	
Total number of lines of therapy received, *n* (%)			0.4
1	319 (72)	644 (70)	
2	85 (19)	171 (19)	
3+	38 (9)	100 (11)	
Class of 2nd line therapy received, *n* (%)			<0.001
Chemotherapy alone	48 (39)	78 (29)	
IO-Chemo combination	23 (19)	23 (8)	
Immunotherapy alone	21 (17)	18 (7)	
Other	31 (25)	152 (56)	

^a^Characteristics were compared between cohorts using a Wilcoxon rank-sum test for continuous measures and a chi-squared test or Fisher’s exact test for categorical measures.

^b^Includes stages IIIB, IIIC, IV, IVA, and IVB.

^c^Assessed on or up to 30 days before treatment start date.

### Gene-level alterations associated with survival in immunotherapy-treated patients

We analyzed genes altered in 5% or more of tumors in either treatment cohort. The prevalence of alterations in *STK11* (11% vs 28%) and *SMARCA4* (4% vs 12%) was lower in the mono-IO cohort compared to the chemo-IO cohort, while the prevalence of alterations in *RBM10* was higher in the mono-IO cohort compared to the chemo-IO cohort (12% vs 7%) (Fig. 1b, Supplementary Fig. 1, FDR *p*-value < 0.05). The observation that *STK11* alterations are more frequent among patients treated with chemo-IO is consistent with the known relationship between *STK11* and PD-L1 status^6^.

**Fig. 2:**
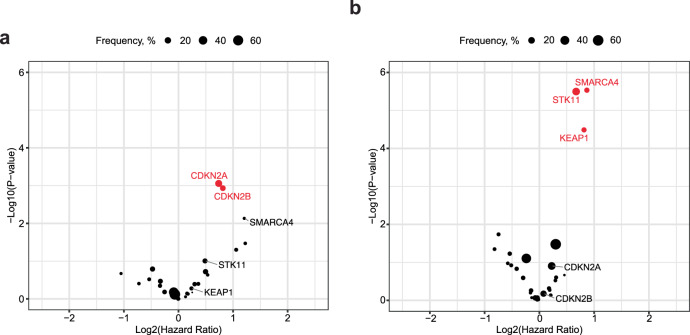
Genomic determinants of immune checkpoint inhibitor response. Volcano plot representing genes associated with survival in the **a** mono-IO cohort and **b** chemo-IO cohort. Significant genes after Benjamini–Hochberg correction (FDR *p*-value < 0.05) are colored in red.

We next assessed associations between gene-level alterations and overall survival. In the mono-IO cohort, 208 (47%) deaths occurred with a median follow-up of 8.3 months and in the chemo-IO cohort 457 (50%) deaths occurred with a median follow-up time of 8.2 months. In the mono-IO cohort, tumors with alterations in *CDKN2A* (HR = 1.7 [1.2–2.2]) or *CDKN2B* (HR = 1.8 [1.3–2.5]) were associated with worse survival compared to the corresponding wild-type tumors (Fig. 2a, FDR *p*-value < 0.05). Interestingly, this association was not observed in the chemo-IO cohort (*CDKN2A* HR = 1.2 [1.0–1.4], uncorrected p-value=0.1; *CDKN2B* HR = 1.1 [0.8–1.3], uncorrected *p*-value = 0.7). Among chemo-IO treated patients, *STK11*, *SMARCA4*, and *KEAP1* altered tumors were associated with worse survival compared to the corresponding wild-type tumors (Fig. 2b, FDR *p*-value < 0.05). While *SMARCA4* did not pass the statistical significance threshold in the mono-IO cohort, the effect size was similar to the chemo-IO cohort (mono-IO HR = 2.3 [1.3–4.2], *p*-value = 0.007, FDR *p*-value = 0.07; chemo-IO HR = 1.8 [1.4–2.3], *p*-value < 0.001).

### Gene deletions on chromosome 9p21.3 are negative predictors of clinical benefit from mono-immunotherapy in patients with NSCLC

**Fig. 3:**
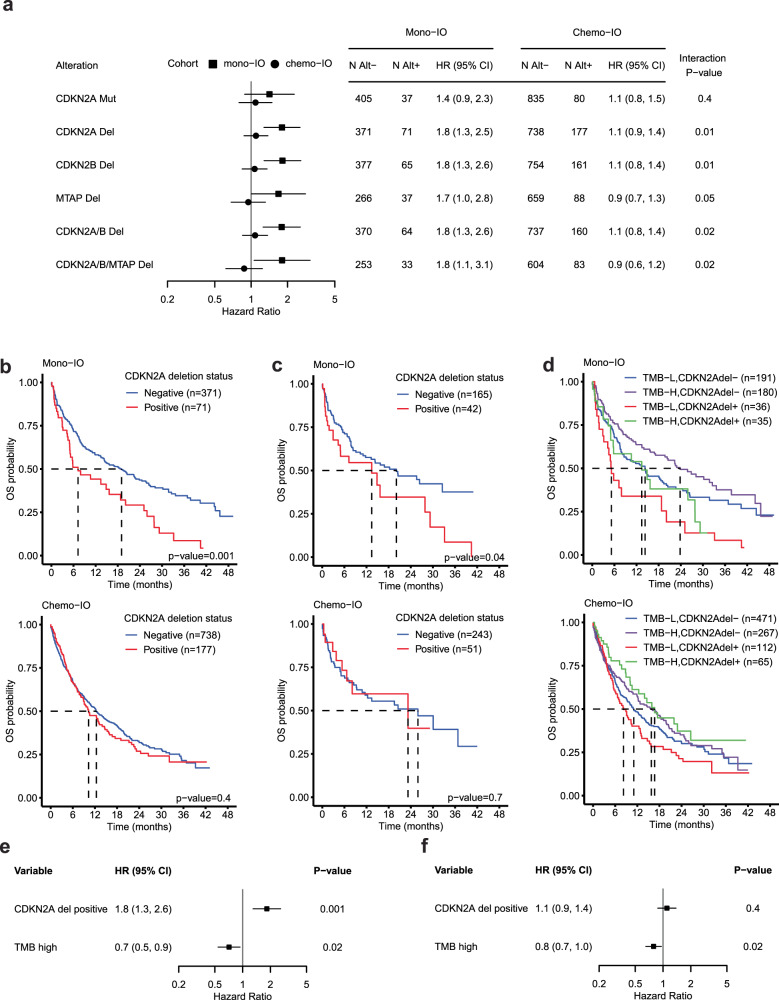
Deletions at chromosome 9p21.3 predict poor response to single-agent immunotherapy. **a** Forest plot of hazard ratios and 95% CIs for overall survival according to specific gene alterations at chromosome 9p21.3. **b**–**d** Kaplan–Meier plot of overall survival for mono-IO (top) and chemo-IO (bottom) treated patients according to *CDKN2A* deletion status. For **c**, analyses are restricted to PD-L1-positive patients. In **d**, overall survival is further stratified by TMB status. Forest plot of hazard ratios and 95% CIs for overall survival from multivariable model for **e** mono-IO and **f** chemo-IO treated patients. TMB-high (≥10 mut/Mb), TMB-low (<10 mut/Mb).

We next investigated whether different types of alterations at chromosome 9p21.3 (*CDKN2A*, *CDKN2B*, *MTAP*) were associated with differential survival after immunotherapy. We considered deleterious short variants and indels, truncating rearrangements, and homozygous deletions (referred to as deletions, see “Methods”). Our findings were most pronounced for *CDKN2A* deletion (mono-IO HR = 1.8 [1.3–2.5], *p*-value = 0.001; chemo-IO HR = 1.1 [0.9–1.4]; *p*-value = 0.4) and *CDKN2B* deletion (mono-IO HR = 1.8 [1.3–2.6], *p*-value = 0.001; chemo-IO HR = 1.1 [0.8–1.4], *p*-value = 0.6) (Fig. 3a). In an interaction model, we found a statistically significant interaction between *CDKN2A* deletion and treatment, and *CDKN2B* deletion and treatment (Fig. 3a, interaction *p*-value = 0.01). *CDKN2A* is also commonly mutated; however, this alteration had less of an association with survival (Fig. 3a). *MTAP* deletion-positive tumors were associated with worse survival in the mono-IO cohort but not the chemo-IO cohort compared to deletion-negative tumors (mono-IO HR = 1.7 [1.0–2.8], *p*-value = 0.05; chemo-IO HR = 0.9 [0.7–1.3], *p*-value = 0.7), although this gene was not baited on all samples which reduced statistical power. Co-deletion of *CDKN2A*, *CDKN2B*, and *MTAP* was similarly predictive of poor survival in mono-IO treated patients (Fig. 3a). In total, these findings suggests that patients whose tumors harbor a deletion on 9p21.3 may have worse outcomes when treated with a single-agent checkpoint inhibitor.

To investigate these survival findings in more detail, we chose to focus on patient subgroups defined by *CDKN2A* deletion given the slightly higher patient count and equivalent effect size as *CDKN2B*. In the mono-IO cohort, patients with *CDKN2A* deletion-positive (*CDKN2A* del+) tumors had decreased mOS compared to *CDKN2A* deletion-negative (*CDKN2A* del−) tumors (Fig. 3b (top), *CDKN2A* del+ mOS (months) = 7.2 [5.0–20.1]; *CDKN2A* del− mOS (months) = 19.0 [14.3–24.5]). In the chemo-IO cohort, median OS was 10.3 and 12.4 months for *CDKN2A* del+ and *CDKN2A* del− tumors respectively (Fig. 3b (bottom)). We also investigated the impact of PD-L1 and TMB, known predictors of immunotherapy response, on these results. When restricting to patients with PD-L1 + tumors, the relationship between *CDKN2A* deletion and survival in the mono-IO cohort remained (Fig. 3c, *p*-value = 0.04). TMB-high tumors (≥10 mut/Mb) were associated with better survival compared to TMB-low tumors (<10 mut/Mb) in both the mono-IO cohort (HR = 0.7 [0.6–1.0], *p*-value = 0.02) and the chemo-IO cohort (HR = 0.8 [0.7–1.0], *p*-value = 0.02). Overall, the TMB-high, *CDKN2A* del− group had the longest mOS of 23.8 [17.2–33.6] months, while the TMB-low, *CDKN2A* del+ group has the shortest mOS of 5.2 [3.1–25.1] months for patients treated with mono-IO (Fig. 3d). Among patients treated with mono-IO, the hazard ratio comparing *CDKN2A* del+ vs *CDKN2A* del− tumors was 1.9 [1.2, 3.1] and 1.7 [1.1, 2.9] for TMB-low and TMB-high patient populations respectively. Furthermore, adjusting for TMB status did not alter the CDKN2A deletion findings (Fig. 3e, f).

We applied a multivariable analysis to investigate whether *CDKN2A* could serve as a predictive biomarker independent of clinical and tumor characteristics. Patient characteristics were largely similar between *CDKN2A* del+ and *CDKN2A* del− patient populations (Supplementary Table 1). TMB and PD-L1 were not different between the cohorts (Supplementary Table 1). After multivariable adjustment for clinical and tumor features (see “Methods”), *CDKN2A* deletion remained predictive of survival after mono-IO treatment with an adjusted hazard ratio of 1.9 [1.3–2.7] and 1.1 [0.8–1.4] for mono-IO and chemo-IO cohorts respectively.

We evaluated time to next treatment (TTNT) as a proxy for progression. *CDKN2A* del− tumors has significantly reduced TTNT compared to *CDKN2A* del+ tumors in the mono-IO cohort (*p*-value = 0.03) but not in the chemo-IO cohort (*p*-value = 0.6) (Supplementary Fig. 2). Mono-IO treated patients with *CDKN2A* del+ tumors had a shorter median TTNT (mTTNT) than patients with *CDKN2A* del− tumors (*CDKN2A* del+ mTTNT (months) = 5.8 [4.8–14.9]; *CDKN2A* del− mTTNT (months) = 9.3 [8.0–11.8]). Among chemo-IO treated patients, median TTNT was similar between *CDKN2A* del+ and *CDKN2A* del− groups (*CDKN2A* del− mTTNT (months) = 7.1 [5.7–10.3]; *CDKN2A* del− mTTNT (months) = 7.9 [7.1–9.2]).

Thus, real-world datapoints indicative of progression align with our survival results and in total suggest that *CDKN2A* deletion is a potential biomarker for selecting between mono-IO and chemo-IO regimens in advanced non-squamous NSCLC.

### Biomarker associations with *CDKN2A* deletion

**Fig. 4:**
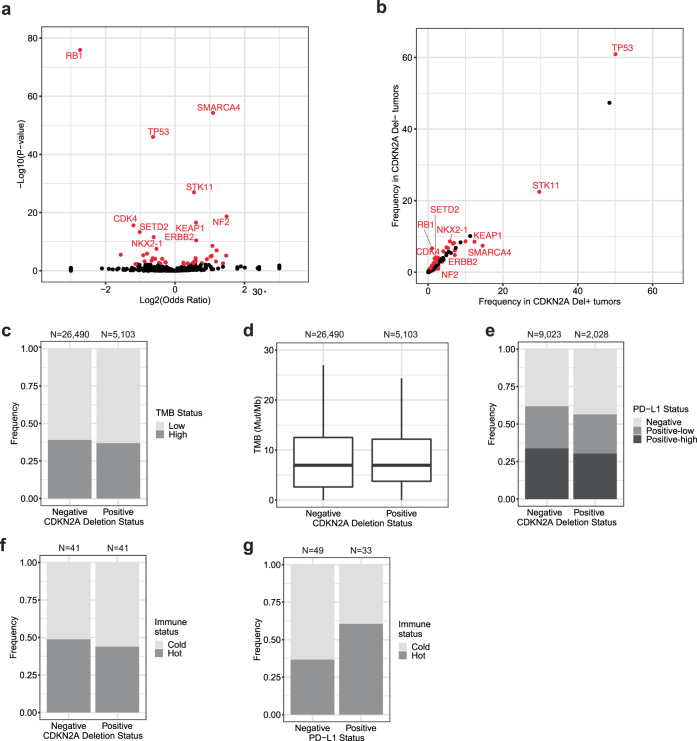
Biomarker associations with *CDKN2A* deletion in *EGFR*/*ALK*- lung adenocarcinoma specimens. **a** Volcano plot demonstrating genomic enrichment according to *CDKN2A* deletion status. **b** Scatter plot of gene frequencies in *CDKN2A* del+ tumors versus *CDKN2A* del− tumors. Fisher’s exact test was used to calculate the odds ratio and *p*-value for the association between gene alteration and *CDKN2A* deletion. Genes with a positive odds ratio are more frequently altered in *CDKN2A* del+(versus *CDKN2A* del−) samples and genes with a negative odds ratio are more frequently altered in *CDKN2A* del− (versus *CDKN2A* del+) samples. Significant genes after Benjamini–Hochberg correction (FDR *p*-value < 0.05) are colored in red. The top ten genes according to *p*-value are labeled. *CDKN2B* and *MTAP* are not included. **c** Bar chart showing frequency of TMB-high (≥10 mut/Mb) and TMB-low (<10 mut/Mb) tumors by *CDKN2A* deletion status. **d** Box plot of TMB values by *CDKN2A* deletion status. The boxplot shows the 1st and 3rd quartiles (upper and lower bounds), 2nd quartile (center), and minimum and maximum values (1.5*interquartile range, whiskers). Outliers are excluded. **e** Bar chart showing frequency of PD-L1-positive and PD-L1-negative tumors by *CDKN2A* deletion status. **f** Bar chart showing frequency of immune hot and cold tumors by *CDKN2A* deletion status. **g** Bar chart showing frequency of immune hot and cold tumors by PD-L1 status. PD-L1 negative (<1% TPS), PD-L1 positive-low (1–49% TPS), and PD-L1 positive-high (≥50 TPS).

We sought to characterize the genomic landscape associated with *CDKN2A* deletion in *EGFR/ALK*- lung adenocarcinoma specimens using the Foundation Medicine genomic dataset. Among 31,600 tumor samples (12,051 with PD-L1 IHC), 5103 (16%) were *CDKN2A* del+ and 26,497 (84%) were *CDKN2A* del−. *CDKN2A* del+ tumors were highly enriched for *CDKN2B* and *MTAP* deletion alterations. Additional genes enriched in *CDKN2A* del+ tumors included *SMARCA4*, *STK11*, *NF2*, *KEAP1*, whereas alterations in *RB1*, *TP53*, *CDK4*, *SETD2*, *NKX2-1*, *ERBB2* were enriched in *CDKN2A* del− tumors (Fig. 4a, b; FDR *p*-value < 0.05).

We next explored the relationship between *CDKN2A* deletion and TMB and PD-L1. The median TMB value was 7.5 mut/Mb in both *CDKN2A* del+ and *CDKN2A* del− tumors (Fig. 4d). 37% of *CDKNA* del+ tumors were TMB-high (≥10 mut/Mb) compared to 39% of *CDKN2A* del− tumors, which was statistically significant despite only a 2% difference (Fig. 4c, *p*-value = 0.003). Similarly, PD-L1 positivity was observed in 57% of *CDKN2A* del+ and 62% of *CDKN2A* del− tumors, which was statistically significant despite the small effect size (Fig. 4e, *p*-value < 0.001).

We also investigated the relationship between *CDKN2A* deletion and the presence or absence of TILs in the tumor microenvironment. A blinded pathologist evaluated 82 samples for immune cell infiltration (50:50 *CDKN2A* del+:del−). Overall, 38 (46%) tumors were categorized as hot (immune-inflamed) and 44 (54%) tumors were categorized as cold (immune-excluded/immune-desert). The percentage of hot/inflamed tumors was similar in both groups at 44% and 49% for *CDKN2A* del+ and *CDKN2A* del− tumors respectively (Fig. 4f, *p*-value = 0.8). As expected, we did observe a higher percentage of hot/inflamed tumors among tumor specimens with PD-L1+ tumor expression compared to PD-L1− tumor expression. 61% of PD-L1+ tumors were inflamed compared to 37% of PD-L1− tumors (Fig. 4g, *p*-value = 0.04). These data suggest that *CDKN2A* loss is not associated with immune infiltration in the tumor microenvironment.

### *CDKN2A* deletion is associated with worse survival following first-line immunotherapy treatment across various cancer types

**Fig. 5:**
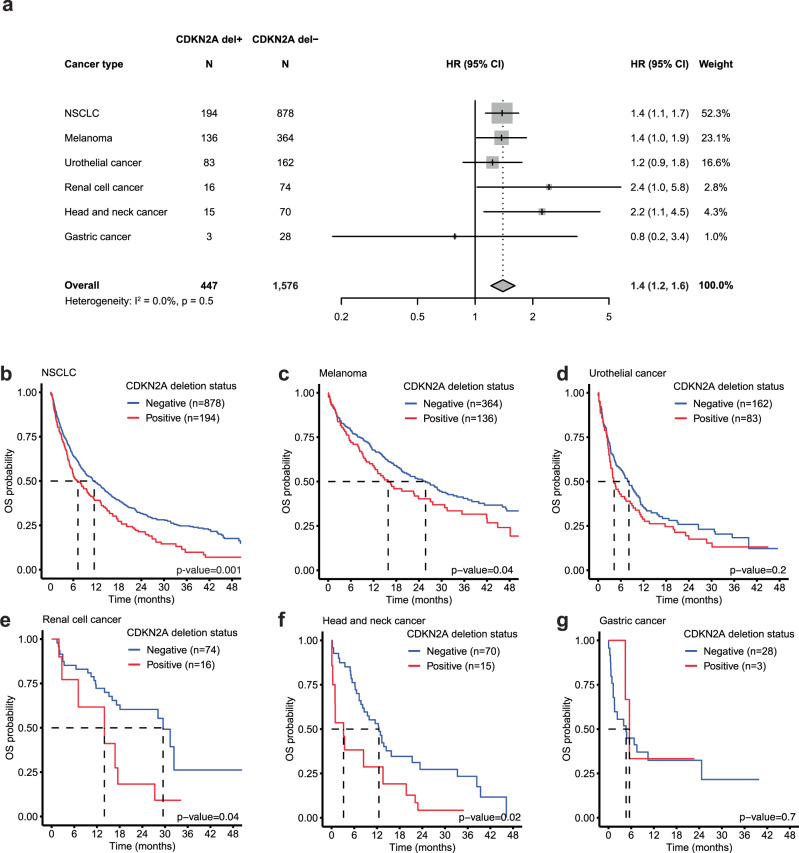
*CDKN2A* deletion is associated with worse immune checkpoint inhibitor response across multiple cancer types. **a** Forest plot of hazard ratios and 95% CIs of overall survival according to *CDKN2A* deletion status in immune checkpoint inhibitor-treated patients across cancer types. Hazard ratios and 95% CIs from each study were used to calculate a pooled HR. The pooled estimate was calculated using a random-effects meta-analysis. Statistical heterogeneity was assessed using the Cochran’s Q test and *I*^2^ statistic. **b**–**g** Kaplan–Meier plot of overall survival according to *CDKN2A* deletion status in immune checkpoint inhibitor-treated patients with **b** NSCLC, **c** melanoma, **d** urothelial cancer, **e** renal cell cancer, **f** head and neck cancer, and **g** gastric cancer.

Deletions at 9p21.3 are common in multiple cancer types. To explore the generalizability of our findings, we examined the association between *CDKN2A* deletion and survival following first-line immunotherapy treatment across various cancer types in CGDB including NSCLC (squamous and non-squamous), melanoma, urothelial cancer, renal cell cancer, head and neck cancer, and gastric cancer. Across cancers, patients in the *CDKN2A* del+ group had shorter survival, which was statistically significant in NSCLC, melanoma, renal cell cancer, and head and neck cancer (Fig. 5a, b; *p*-value < 0.05). A significant association was observed for non-squamous NSCLC patients (HR = 1.5 [1.2–1.9]); however, no association was observed for those with squamous NSCLC (HR = 1.1 [0.8, 1.6]). In a meta-analysis of all six cancer types, the overall hazard ratio was 1.4 [1.2–1.6] (Fig. 5a). Excluding NSCLC, the largest immunotherapy treated patient population, the association between *CDKN2A* deletion and survival remained (HR = 1.4 [1.1–1.8]).

## Discussion

There are multiple first-line treatment options for patients with PD-L1+, *EGFR/ALK*-, non-squamous NSCLC including monotherapy targeting PD-1/PD-L1, as well as blockade of PD-1/PD-L1 in combination with chemotherapy or CTLA-4 blockade. The pivotal trials for these regimens all used platinum doublet chemotherapy as the comparator, making it difficult to choose between these different regimens^1^. Current guidelines for immunotherapy treatment selection are based on PD-L1 tumor expression levels and physicians often use patient health as another clinical factor when selecting therapies (see age and ECOG biases in Table 1)^1^.

In this work, we utilized a large cohort of lung cancer patients in the real-world setting to assess genomic markers that may have utility in selecting mono-IO vs chemo-IO regimens in non-squamous NSCLC. Patients with tumors that harbored deletions at the chromosome locus 9p21.3 (*CDKN2A*/*CDKN2B*/*MTAP*) had statistically significant worse outcomes when treated with mono-IO but not chemo-IO, which suggests these patients may benefit from more aggressive therapy. Our finding that PD-L1 and *CDKN2A* status are uncorrelated provides an opportunity to combine these biomarkers in making treatment decisions. A meta-analysis across six different cancer types highlights the potential pan-cancer relevance of *CDKN2A* deletion as a negative indicator of clinical benefit after immunotherapy.

We assessed multiple potential biomarkers involving genes at 9p21.3. Deletions at this locus almost always include both *CDKN2A* and *CDKN2B*, which explains why deletion biomarkers for each of these genes produced equivalent results. *MTAP* is also frequently lost; however, it was not baited for in all test samples which reduced statistical power. *CDKN2A* is also frequently mutated, but the effect of *CDKN2A* mutation on outcome was less and not significant. While we defined the biomarker as *CDKN2A* deletion for simplicity, these findings suggest that it is a deletion in 9p21.3, which likely affects multiple genes, that is the relevant biomarker. In addition to the genes evaluated, interferon (IFN) and related pathway genes are also clustered in the 9p21.3 chromosome region and have been implicated in the antitumor immune response^7^. IFN genes are not included on our targeted assay so a direct association between these genes and outcomes cannot be examined.

Recent studies have also highlighted the potential utility of 9p21.3 and *CDKN2A* as a biomarker of immunotherapy outcomes^8–16^. Banchereau et al. showed a significant association between elevated expression of *CDKN2A* and improved overall survival following mono-immunotherapy treatment for urothelial cancer and NSCLC^12^. Gene expression is regulated by a multitude of factors; however, it is likely that samples with a deletion at 9p21.3 lack expression of *CDKN2A*. In a pan-cancer study, Han et al. found significantly lower response rates to anti-PD-1/PD-L1 monotherapy for patients with solid tumors that harbored a 9p21 loss, including melanoma, urothelial, and NSCLC^14^. The authors showed 9p21 loss was associated with reduced abundance of TILs inferred from gene expression data and lower PD-L1 positivity; however, the strength of this finding varied by cancer type and patient cohort. We did not find evidence that 9p21 loss is associated with a “cold” tumor-immune phenotype in our study which was focused on *EGFR*/*ALK*- lung adenocarcinoma specimens and acknowledge that there are potentially significant tumor type differences in this relationship that need to be explored. Adib et al. demonstrated an association between *CDKN2A* loss of function alterations and poor outcomes in ICI-treated patients in urothelial and melanoma cancer^13^. They did not observe an association with NSCLC which may be due to several differences (1) the inclusion of mutations in their biomarker definition, which we observed had a weaker effect than deletions, (2) the fraction of squamous cancers in the dataset since we observed no effect of *CDKN2A* deletion in squamous lung cancer, (3) the inclusion of *EGFR/ALK*+ patients which we excluded, or (4) inclusion of multiple lines of therapy since we analyzed only first-line patients and found the effect weakened in later lines (data not shown). Gutiontov et al. demonstrated an association between *CDKN2A* loss-of-function and worse outcomes among immunotherapy treated patients with advanced NSCLC^15^. In this study, immunotherapy treatment included both immunotherapy alone or in combination with chemotherapy; however, the authors noted that the effect of *CDKN2A* loss-of-function on disease control rate was observed in patients receiving mono-immunotherapy and not for those who received combination therapy which is in agreement with our findings. Finally, Alhalabi et al. demonstrated that MTAP deficiency commonly due to 9p21 loss was associated with inferior outcomes in a cohort of patients with metastatic urothelial cancer treated with immunotherapy and standard-of-care chemotherapy^16^. We did not observe an association between 9p21 deletions (*CDKN2A*, *CDKN2B*, *MTAP*) and survival in non-squamous NSCLC patients treated with chemotherapy-immunotherapy combination treatment. It has been shown that conventional chemotherapy enhances the effects of immunotherapy by inducing immunogenic cell death and inducing an antitumor immune response^17^. One limitation of our study is that our findings may be due to pemetrexed specifically and may not apply to the broad category of chemotherapy-immunotherapy combinations. Further investigation is required to resolve these differences and determine the best *CDKN2A-*associated biomarker for predicting patient benefit from immunotherapy.

Our data support 9p21.3 loss as an immunotherapy resistance biomarker with the potential to inform immunotherapy treatment decisions for non-squamous NSCLC patients. While previous studies have focused on immunotherapy-only cohorts, our study, which compares mono-IO vs chemo-IO treatment options, demonstrated a reduced effectiveness of mono-IO in CDKN2A del+ patients and identified a potential solution in chemo-IO. Additional studies are needed to investigate whether patients whose tumor harbors a 9p21.3 deletion benefit from chemo-IO strategies outside of non-squamous NSCLC.

## Methods

### Genomic profiling

Genomic data were collected using a tissue-based, targeted comprehensive genomic profiling (CGP) assay (FoundationOne or FoundationOne CDx) in a Clinical Laboratory Improvement Amendments (CLIA)-certified, College of American Pathologists (CAP)-accredited, New York State-approved laboratory (Foundation Medicine, Inc.), as previously described^18^. DNA was extracted from formalin-fixed paraffin-embedded (FFPE) tumor tissue specimens and underwent adaptor ligated hybridization capture for 324 genes in FoundationOne CDx (310 all coding exons and select introns in 34 genes) or 404 genes in FoundationOne (395 all coding exons and select introns in 31 genes). Libraries were sequenced to a median unique coverage depth of >500X. Analysis for genomic alterations, including short variant alterations (base substitutions, insertions, and deletions), copy-number alterations (amplifications and homozygous deletions), as well as gene rearrangements, was performed as previously described^18,19^. Hemizygous or shallow deletions were excluded from this analysis. TMB was defined as the number of non-driver somatic coding mutations per megabase of genome sequenced^20^.

### Histology

PD-L1 status was determined through immunohistochemistry (IHC) performed on FFPE tissue sections with 22C3 (Dako/Agilent, Santa Clara, CA, USA). A board-certified pathologist determined the tumor proportion score (TPS) for each sample as defined by the assay package insert for use as a companion diagnostic. The TPS is the proportion of tumor cells exhibiting linear membranous staining out of all tumor cells and is reported as a percentage (0–100%). PD-L1 expression was summarized as negative (<1% TPS) or positive (≥1% TPS). The pathology laboratory established performance characteristics for this assay per the requirements of the Clinical Laboratory Improvement Amendments (CLIA ’88) and in accordance with College of American Pathologists (CAP) checklist requirements and guidance.

Evaluation of tumor-infiltrating lymphocytes (TILs) was performed on a random sample of 41 *CDKN2A* deletion-negative and 41 *CDKN2A* deletion-positive lung adenocarcinoma resection specimens by a board-certified pathologist blinded to *CDKN2A* status. Hematoxylin and eosin-stained section slides were categorized as hot (immune-infiltrated) or cold (immune-excluded/immune-desert).

### Data sources and patient cohorts

#### Flatiron Health-Foundation Medicine Clinico-Genomic Database (FH-FMI CGDB)

This study utilized real-world data from the nationwide (US-based) de-identified FH-FMI CGDB (data collected through December 31, 2020). Retrospective longitudinal clinical data were derived from electronic health record (EHR) data, comprising patient-level structured and unstructured data curated via technology-enabled abstraction, and were linked to genomic data derived from FMI CGP tests by de-identified, deterministic matching^21,22^. Institutional Review Board approval of the study protocol was obtained prior to study conduct and included a waiver of informed consent based on the observational, non-interventional nature of the study (WCG IRB, Protocol No. 420180044).

For the primary analysis, patients were selected if they received first-line treatment with pembrolizumab monotherapy (*n* = 442) or pembrolizumab plus cisplatin/carboplatin and pemetrexed (*n* = 915) (Fig. 1a). For the pan-cancer analysis, patients were selected if they received an immunotherapy-exclusive regimen containing anti-PD-1/PD-L1 (pembrolizumab, nivolumab, cemiplimab, atezolizumab, durvalumab, avelumab) and/or anti-CTLA-4 (ipilimumab) agent as a first-line treatment for advanced or metastatic disease. Cancer types with sufficient data for further analysis included NSCLC (*n* = 1072), melanoma (*n* = 500), urothelial cancer (*n* = 245), renal cell cancer (*n* = 90), head and neck cancer (*n* = 85), and gastric cancer (*n* = 31).

#### Foundation medicine genomic database

The Foundation Medicine genomic database consists of tumor specimens that underwent FMI CGP (sequenced through December 31, 2020). For this study, we identified 31,600 *EGFR/ALK*-, lung adenocarcinoma specimens, a subset of which (*n* = 11,051) underwent PD-L1 IHC. Approval for this study, including a waiver of informed consent and a HIPAA waiver of authorization, was obtained from the Western Institutional Review Board (Protocol No. 20152817). The Institutional Review Board granted a waiver of informed consent under 45 CFR § 46.116 based on review and determination that this research meets the following requirements: (i) the research involves no more than minimal risk to the subjects; (ii) the research could not practicably be carried out without the requested waiver; (iii) the waiver will not adversely affect the rights and welfare of the subjects.

### Statistical analysis

Overall survival (OS) was defined from the date of first-line treatment to date of death with censoring at the last known visit date. TTNT was defined from the start date of first-line treatment to start date of a subsequent line of therapy or death date if subsequent therapy was not initiated with censoring at the last known visit date. We accounted for left truncation time from the treatment date to FMI test date. Survival curves and estimates of median survival time were generated using the Kaplan–Meier method. Univariable Cox proportional hazards regression was used to estimate hazard ratios (HRs) and 95% confidence intervals (CIs) for the association between genomic alterations and survival, with statistical significance determined by the Wald test. For select findings, an alteration x first-line treatment interaction term was included in the Cox model. Multivariable analyses adjusted for TMB, as well as age, gender, race, practice type, smoking history, advanced stage at diagnosis, ECOG performance status (PS), tumor type, biopsy site, and *TP53* and *KRAS* alteration status.

Two-sided *p*-values are reported. *P*-values were adjusted for multiple comparisons using the Benjamini–Hochberg false discovery (FDR) method. Analyses were performed using R version 3.6.2.

### Reporting summary

Further information on research design is available in the Nature Research Reporting Summary linked to this article.

## Supplementary information

Supplementary Information

REPORTING SUMMARY

## Data Availability

Consented data that can be released are included in the article and its supplementary files. Patients were not consented for the release of underlying sequence data. Academic researchers can gain access to Foundation Medicine data in this study by contacting the corresponding author and filling out a study review committee form. You and your institution will be required to sign a data transfer agreement.

## Code availability

No custom code was used in this study.
