# Supplementary material for: Deletions on 9p21 are associated with worse outcomes after anti-PD-1/PD-L1 monotherapy but not chemoimmunotherapy
Source: NPJ Precis Oncol. 2022 Jun 23;6:44. doi: 10.1038/s41698-022-00286-4 (PMC9225995; doi:10.1038/s41698-022-00286-4)

Supplementary Tables

Supplementary Table 1. Demographic and clinical characteristics by *CDKN2A* deletion status

		1st line mono-IO				1st line chemo-IO		
		***CDKN2A* del+ (n=71)**	***CDKN2A* del- (n=371)**	**P-value[1]**		***CDKN2A* del+ (n=177)**	***CDKN2A* del- (n=738)**	**P-value[1]**
**Age at treatment start, years, median (range)**		74 (40, 85)	72 (38, 85)	0.03		69 (36, 85)	68 (33, 85)	0.8
**Gender, n (%)**				0.3				0.01
	Female	36 (51)	215 (58)			67 (38)	360 (49)	
	Male	35 (49)	156 (42)			110 (62)	378 (51)	
**Race, n (%)**				0.7				0.06
	Asian	<5	<5			<5	7 (1)	
	Black or African American	<5	18 (5)			13 (7)	47 (6)	
	Other	11 (15)	54 (15)			17 (10)	127 (17)	
	White	52 (73)	270 (73)			121 (68)	489 (66)	
	Missing	6 (8)	25 (7)			22 (12)	68 (9)	
**Smoking history, n (%)**				1.0				0.6
	Yes	67 (94)	351 (95)			152 (86)	670 (91)	
	No	4 (6)	20 (5)			25 (14)	68 (9)	
**Practice type, n (%)**				0.6				0.8
	Academic	6 (8)	25 (7)			11 (6)	39 (5)	
	Community	65 (92)	346 (93)			166 (94)	699 (95)	
**Advanced stage at diagnosis[2], n (%)**				0.2				0.9
	Yes	53 (75)	239 (64)			148 (84)	606 (82)	
	No	17 (24)	126 (34)			27 (15)	123 (17)	
	Not reported	1 (1)	6 (2)			2 (1)	9 (1)	
**ECOG performance status[3], n (%)**				0.6				0.07
	0	10 (14)	78 (21)			48 (27)	203 (28)	

1	29 (41)	134 (36)			59 (33)	273 (37)	
2+	19 (27)	88 (24)			39 (22)	105 (14)	
Missing	13 (18)	71 (19)			31 (18)	157 (21)	
**Disease type, n (%)**			0.01				0.5
Lung adenocarcinoma	48 (68)	298 (80)			150 (85)	623 (84)	
Lung non-small cell lung carcinoma (nsclc) (nos)	12 (17)	52 (14)			15 (8)	78 (11)	
Other	11 (15)	21 (6)			12 (7)	37 (5)	
**Tissue of origin, n (%)**			0.2				0.007
Lung	26 (37)	185 (50)			66 (37)	360 (49)	
Lymph node	10 (14)	50 (13)			21 (12)	104 (14)	
Brain	9 (13)	28 (8)			13 (7)	39 (5)	
Bone	2 (3)	17 (5)			18 (10)	37 (5)	
Other/unknown	24 (34)	91 (25)			59 (33)	198 (27)	
**TMB, n (%)**			1.0				1.0
Low (<10 mut/Mb)	36 (51)	191 (51)			112 (63)	471 (64)	
High (≥10 mut/Mb)	35 (49)	180 (49)			65 (37)	267 (36)	
**PD-L1 expression, n (%)**			0.5				0.2
No (<1% TPS)	1 (2)	15 (8)			44 (46)	140 (37)	
Low (1-49% TPS)	7 (16)	26 (14)			29 (31)	141 (37)	
High (≥50% TPS)	35 (81)	139 (77)			22 (23)	102 (27)	
Missing	28	191			82	355	
**Total number of lines of therapy received, n (%)**			0.9				1.0
1	50 (70)	269 (73)			124 (70)	520 (70)	
2	14 (20)	71 (19)			33 (19)	138 (19)	
3+	7 (10)	31 (8)			20 (11)	80 (11)	
**Class of 2nd line therapy received, n (%)**			1.0				0.06

	Chemotherapy alone	8 (38)	40 (39)			15 (28)	63 (29)	
	IO-Chemo combination	4 (19)	19 (19)			9 (17)	14 (6)	
	Immunotherapy alone	4 (19)	17 (17)			1 (2)	17 (8)	
	Other	5 (24)	26 (25)			28 (53)	124 (57)	

[1]Characteristics were compared between cohorts using a Wilcoxon rank-sum test for continuous measures and a chi-squared test or Fisher's exact test for categorical measures.
[2]Includes stages IIIB, IIIC, IV, IVA, IVB.
[3]Assessed on or up to 30 days before treatment start date.

Supplementary Figures

Supplementary Figure 1. Genomic characterization of the clinical cohort. (a-b) Bar chart of the prevalence of gene alterations by alteration type for patients in the (a) mono-IO and (b) chemo-IO cohort. (c) Oncoprint plot of prevalent genomic alterations in the mono-IO and chemo-IO cohort.

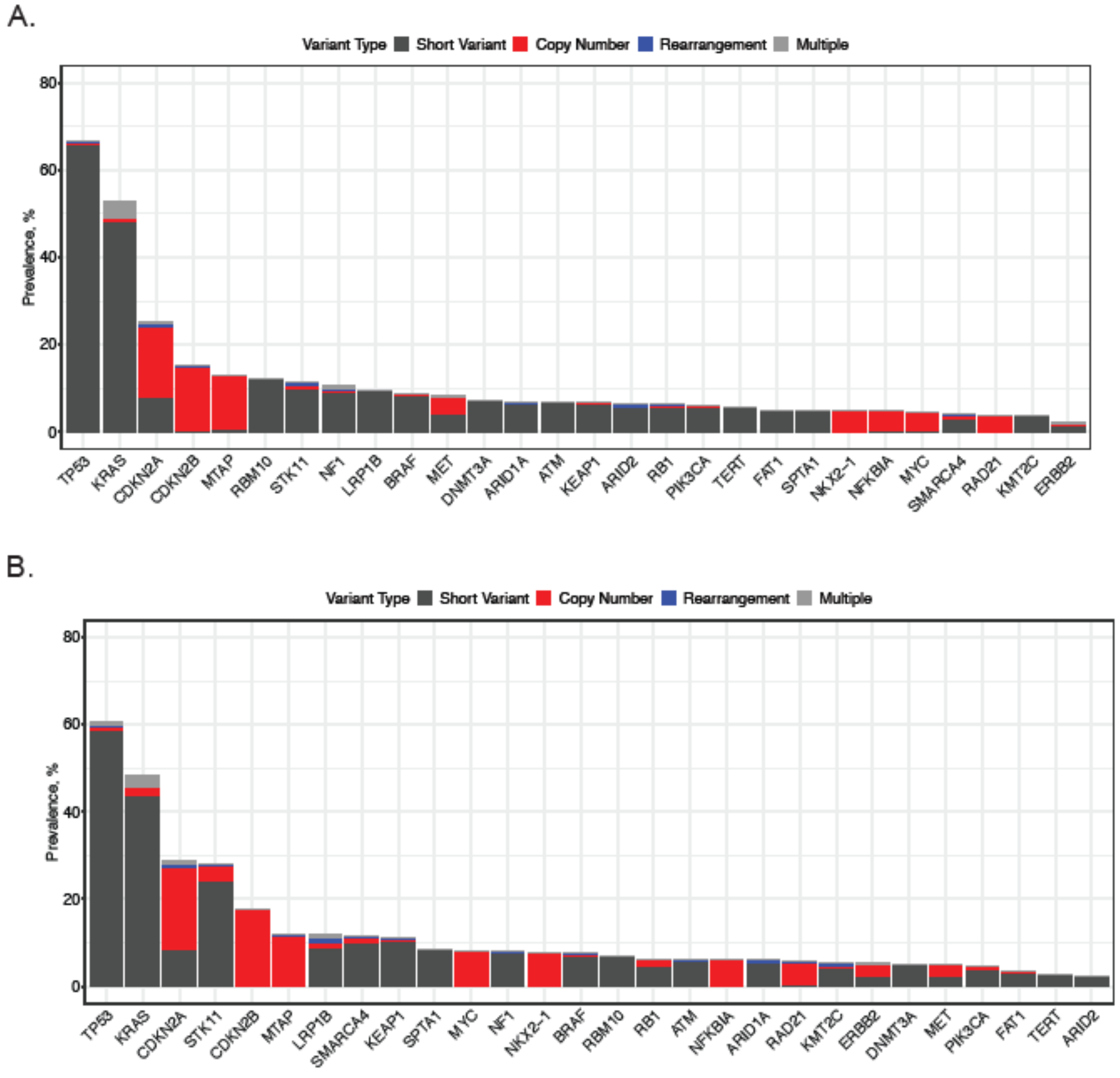

C.

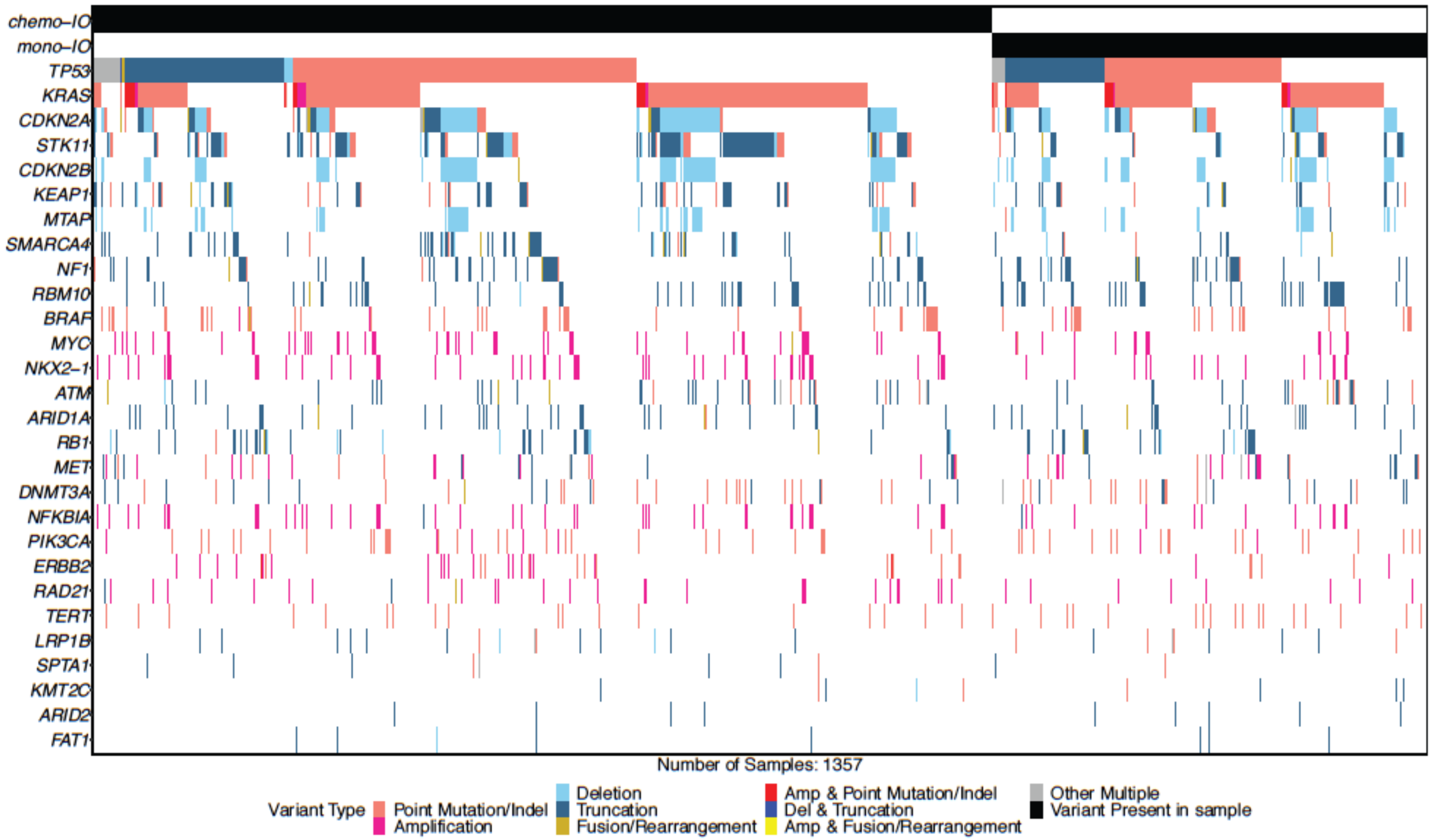
chemo–IO
mono–IO
TP53
KRAS
CDKN2A
STK11
CDKN2B
KEAP1
MTAP
SMARCA4
NF1
RBM10
BRAF
MYC
NKX2–1
ATM
ARID1A
RB1
MET
DNMT3A
NFKBIA
PIK3CA
ERBB2
RAD21
TERT
LRP1B
SPTA1
KMT2C
ARID2
FAT1
Number of Samples: 1357
Variant Type
Point Mutation/Indel
Amplification
Deletion
Truncation
Fusion/Rearrangement
Amp & Point Mutation/Indel
Del & Truncation
Amp & Fusion/Rearrangement
Other Multiple
Variant Present in sample

Supplementary Figure 2. *CDKN2A* deletion is associated with lower time to next treatment (TTNT) after first-line immunotherapy. Kaplan-Meier plot of TTNT for mono-IO (left) and chemo-IO (right) treated patients according to *CDKN2A* deletion status.

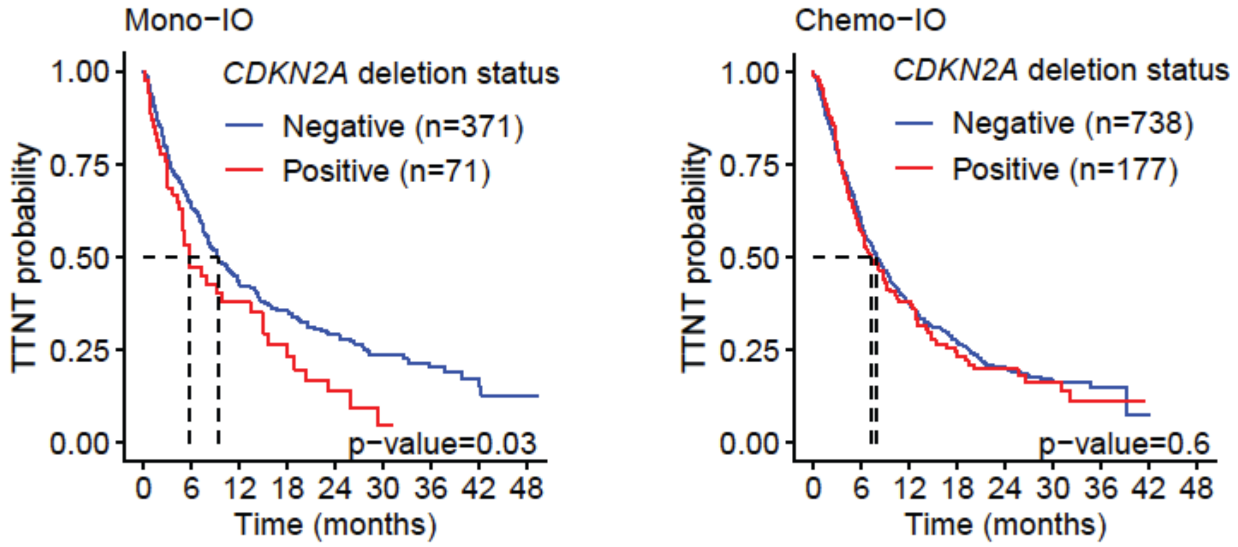

Supplement: Supplementary file 1 — Supplementary Information [file 41698_2022_286_MOESM1_ESM.pdf]
