# Supplementary material for: Deletions on 9p21 are associated with worse outcomes after anti-PD-1/PD-L1 monotherapy but not chemoimmunotherapy
Source: NPJ Precis Oncol. 2022 Jun 23;6:44. doi: 10.1038/s41698-022-00286-4 (PMC9225995; doi:10.1038/s41698-022-00286-4)
Supplement: Supplementary file 2 — REPORTING SUMMARY [file 41698_2022_286_MOESM2_ESM.pdf]

nature portfolio

Corresponding author(s): Lee A. Albacker

Last updated by author(s): Apr 29, 2022

# Reporting Summary

Nature Portfolio wishes to improve the reproducibility of the work that we publish. This form provides structure for consistency and transparency in reporting. For further information on Nature Portfolio policies, see our Editorial Policies and the Editorial Policy Checklist.

## Statistics

For all statistical analyses, confirm that the following items are present in the figure legend, table legend, main text, or Methods section.

| n/a | Confirmed | |
|---|---|---|
| ☐ | ☒ | The exact sample size (*n*) for each experimental group/condition, given as a discrete number and unit of measurement |
| ☐ | ☒ | A statement on whether measurements were taken from distinct samples or whether the same sample was measured repeatedly |
| ☐ | ☒ | The statistical test(s) used AND whether they are one- or two-sided<br>*Only common tests should be described solely by name; describe more complex techniques in the Methods section.* |
| ☐ | ☒ | A description of all covariates tested |
| ☐ | ☒ | A description of any assumptions or corrections, such as tests of normality and adjustment for multiple comparisons |
| ☐ | ☒ | A full description of the statistical parameters including central tendency (e.g. means) or other basic estimates (e.g. regression coefficient) AND variation (e.g. standard deviation) or associated estimates of uncertainty (e.g. confidence intervals) |
| ☐ | ☒ | For null hypothesis testing, the test statistic (e.g. *F*, *t*, *r*) with confidence intervals, effect sizes, degrees of freedom and *P* value noted<br>*Give P values as exact values whenever suitable.* |
| ☒ | ☐ | For Bayesian analysis, information on the choice of priors and Markov chain Monte Carlo settings |
| ☒ | ☐ | For hierarchical and complex designs, identification of the appropriate level for tests and full reporting of outcomes |
| ☐ | ☒ | Estimates of effect sizes (e.g. Cohen's *d*, Pearson's *r*), indicating how they were calculated |

*Our web collection on statistics for biologists contains articles on many of the points above.*

## Software and code

Policy information about availability of computer code

| | |
|---|---|
| Data collection | No software was used for data collection. |
| Data analysis | Analyses were performed using R version 3.6.2. No custom code was used in this study. |

For manuscripts utilizing custom algorithms or software that are central to the research but not yet described in published literature, software must be made available to editors and reviewers. We strongly encourage code deposition in a community repository (e.g. GitHub). See the Nature Portfolio guidelines for submitting code & software for further information.

## Data

Policy information about availability of data

All manuscripts must include a data availability statement. This statement should provide the following information, where applicable:

- Accession codes, unique identifiers, or web links for publicly available datasets
- A description of any restrictions on data availability
- For clinical datasets or third party data, please ensure that the statement adheres to our policy

Consented data that can be released are included in the article and its supplementary files. Patients were not consented for the release of underlying sequence data. Academic researchers can gain access to Foundation Medicine data in this study by contacting the corresponding author and filling out a study review committee form. You and your institution will be required to sign a data transfer agreement.

# Field-specific reporting

Please select the one below that is the best fit for your research. If you are not sure, read the appropriate sections before making your selection.

☒ Life sciences ☐ Behavioural & social sciences ☐ Ecological, evolutionary & environmental sciences

For a reference copy of the document with all sections, see nature.com/documents/nr-reporting-summary-flat.pdf

# Life sciences study design

All studies must disclose on these points even when the disclosure is negative.

**Sample size**

All available advanced non-squamous NSCLC patients without EGFR mutations or ALK rearrangements (EGFR/ALK-) treated with first-line pembrolizumab monotherapy between 2016-2020 (mono-IO cohort, n=442) or first-line pembrolizumab plus chemotherapy between 2017-2020 (chemo-IO cohort, n=915) within the real-world CGDB were chosen for this study.

**Data exclusions**

The NSCLC CGDB included 6,678 patients who had a medical record-confirmed diagnosis of advanced non-squamous NSCLC, received care within the Flatiron Health network between January 2011 and December 2020, and underwent tissue-based CGP by Foundation Medicine during their cancer care. Among these patients, 4,979 had documentation of initiation of at least one line of systemic therapy after advanced diagnosis. This study was limited to advanced non-squamous NSCLC patients whose tumor did not harbor an EGFR short variant or ALK rearrangement, and received first-line treatment with pembrolizumab monotherapy or first-line treatment with pembrolizumab plus pemetrexed and platinum-based chemotherapy.

**Replication**

We examined the association between CDKN2A deletion and survival following first-line immunotherapy treatment across various cancer types in CGDB including NSCLC (squamous and non-squamous), melanoma, urothelial cancer, renal cell cancer, head and neck cancer, and gastric cancer.

**Randomization**

Our study did not utilize randomization.

**Blinding**

Blinding is not relevant for this study since it is a retrospective cohort study.

# Reporting for specific materials, systems and methods

We require information from authors about some types of materials, experimental systems and methods used in many studies. Here, indicate whether each material, system or method listed is relevant to your study. If you are not sure if a list item applies to your research, read the appropriate section before selecting a response.

## Materials & experimental systems

| n/a | Involved in the study | |
|---|---|---|
| ☐ | ☒ | Antibodies |
| ☒ | ☐ | Eukaryotic cell lines |
| ☒ | ☐ | Palaeontology and archaeology |
| ☒ | ☐ | Animals and other organisms |
| ☐ | ☒ | Human research participants |
| ☒ | ☐ | Clinical data |
| ☒ | ☐ | Dual use research of concern |

## Methods

| n/a | Involved in the study | |
|---|---|---|
| ☒ | ☐ | ChIP-seq |
| ☒ | ☐ | Flow cytometry |
| ☒ | ☐ | MRI-based neuroimaging |

## Antibodies

**Antibodies used**

PD-L1 status was determined through immunohistochemistry (IHC) performed on FFPE tissue sections with 22C3 (Dako/Agilent, Santa Clara, CA, USA).

**Validation**

*Describe the validation of each primary antibody for the species and application, noting any validation statements on the manufacturer's website, relevant citations, antibody profiles in online databases, or data provided in the manuscript.*

## Human research participants

Policy information about studies involving human research participants

**Population characteristics**

This study included advanced non-squamous NSCLC patients without EGFR mutations or ALK rearrangements (EGFR/ALK-) treated with first-line pembrolizumab monotherapy between 2016-2020 (mono-IO cohort, n=442) or first-line pembrolizumab plus chemotherapy between 2017-2020 (chemo-IO cohort, n=915) within the real-world CGDB (Figure 1A). Patients in the mono-IO cohort were older (median age: 72 years vs 68 years), more likely to be female (57% vs 47%), and more likely to have a history of smoking (95% vs 90%) compared to patients in the chemo-IO cohort (Table 1). A higher

proportion of mono-IO treated patients had non-advanced stage at initial diagnosis (32% vs 16%) and poor ECOG PS (≥2: 24% vs 16%) compared to chemo-IO treated patients. 49% of patients in the mono-IO cohort had tumors with high TMB (≥10 mut/Mb) compared to 36% of patients in the chemo-IO cohort. Almost all (93%) mono-IO treated patients had PD-L1-positive (PD-L1+) tumors, compared to 62% of chemo-IO treated patients. Median overall survival (mOS) for the mono-IO and chemo-IO cohort was 15.7 [13.4-22.3] months and 12.3 [10.5-14.0] months respectively.

| | |
|---|---|
| Recruitment | Patients were not recruited as this is a retrospective cohort study using real world data. |
| Ethics oversight | WCG IRB |

Note that full information on the approval of the study protocol must also be provided in the manuscript.
